# Phylogeny of Toll-Like Receptor Signaling: Adapting the Innate Response

**DOI:** 10.1371/journal.pone.0054156

**Published:** 2013-01-11

**Authors:** Jeffrey M. Roach, Luigi Racioppi, Corbin D. Jones, Anna Maria Masci

**Affiliations:** 1 Research Computing Center, University of North Carolina, Chapel Hill, North Carolina, United States of America; 2 Department of Medicine, Duke University, Durham, North Carolina; United States of America; 3 Department of Cellular and Molecular Biology and Pathology, University of Naples Federico II, Naples, Italy; 4 Department of Biology, University of North Carolina, Chapel Hill, North Carolina, United States of America; 5 Department of Immunology, Duke University, Durham, North Carolina, United States of America; University of Cambridge, United Kingdom

## Abstract

The Toll-like receptors represent a largely evolutionarily conserved pathogen recognition machinery responsible for recognition of bacterial, fungal, protozoan, and viral pathogen associated microbial patterns and initiation of inflammatory response. Structurally the Toll-like receptors are comprised of an extracellular leucine rich repeat domain and a cytoplasmic Toll/Interleukin 1 receptor domain. Recognition takes place in the extracellular domain where as the cytoplasmic domain triggers a complex signal network required to sustain appropriate immune response. Signal transduction is regulated by the recruitment of different intracellular adaptors. The Toll-like receptors can be grouped depending on the usage of the adaptor, MyD88, into MyD88-dependent and MyD88 independent subsets. Herein, we present a unique phylogenetic analysis of domain regions of these receptors and their cognate signaling adaptor molecules. Although previously unclear from the phylogeny of full length receptors, these analyses indicate a separate evolutionary origin for the MyD88-dependent and MyD88-independent signaling pathway and provide evidence of a common ancestor for the vertebrate and invertebrate orthologs of the adaptor molecule MyD88. Together these observations suggest a very ancient origin of the MyD88-dependent pathway Additionally we show that early duplications gave rise to several adaptor molecule families. In some cases there is also strong pattern of parallel duplication between adaptor molecules and their corresponding TLR. Our results further support the hypothesis that phylogeny of specific domains involved in signaling pathway can shed light on key processes that link innate to adaptive immune response.

## Introduction

The pattern recognition receptors (PRR) of innate immunity form a large class of germ-line encoded receptors implicated in the detection of pathogen associated microbial patterns (PAMPs) and the initiation of downstream signaling cascades. Although these proteins are widely expressed, they play an important role in cell types collectively named antigen presenting cells (APC), such macrophages and dendritic cells, and specialized in sampling and presenting microbial antigens to lymphocyte [1]. The signaling cascades triggered by PRR result in a wide range of biological effects including cytokines release, terminal differentiation, migration, survival or death [2], [3]. This plasticity is largely dependent by extensive crosstalk between PPR-signals and other receptor-coupled pathways, which links PPR canonical cascade with intracellular circuits regulating metabolism, cytoskeleton organization, and cell survival [4]–[10]. Overall this complex network of interactions determines the balance between immune-mediated tissue-protective and tissue-destructive events occurring in the body following PPR stimulation.

Among the best characterized pattern recognition receptors, the Toll-like receptors (TLRs), are named for their homology with the Toll receptor first identified in *Drosophila melanogaster.* Originally isolated in connection with dorsal-ventral developmental patterning [11], the Toll receptor was later shown to play a role in fungal and bacterial resistance [12], [13]. Receptors analogous to the Toll receptor have been found in both vertebrates and invertebrates and represent an ancient and evolutionarily conserved host defense mechanism. In fact, similarities to the Toll receptor have been shown to a lesser degree in proteins coded by the plant resistance genes such as the N gene in *Nicotiana glutinosa* [14], [15].

The evolution of TLR signaling is driven by the need to conserve the capability to recognize specific pathogen signature while at the same time allowing for the development of new platforms on which to build more complex signaling networks drives. To better understand this dynamic and the molecular evolution of TLRs, phylogenetic reconstructions of the TLRs have been calculated from both full-length gene or amino acid sequences [16]–[22]. This work suggests that vertebrate TLRs arose by and ancient gene duplication that has since given rise to two large gene families of TLRs [20] and subsequent evolution of these families resulted from a complex interaction between gene duplication, gene conversion, and co-evolution [21]. The phylogenetic picture of TLRs, however, is complicated by difficulties in predicting leucine-rich repeat structure and in reliably aligning full-length sequences for TLRs of widely divergent species. Most phylogenies have been restricted to the cytoplosmic TIR domain [20]–[22]. Phylogenetic investigations of the TLR signaling pathways have focused on the full sequence phylogeny of individual proteins, including the TLRs, that occur along the NF-κB pathway such as: Pelle, IkB, Rel, and TRAF [16].

Individual protein domains are the palette of structural units from which protein functions are composed [23]–[25]. Thus, phylogenetic investigations at the domain level can reveal aspects of protein evolution that may be confounded at the level of entire protein sequences alone [26]–[28]. Structurally the TLRs are transmembrane proteins with common domain structure reflecting both pattern recognition and downstream signaling functions [29]–[35]. TLR signaling, in particular, takes place through individual domains that induce interactions of entire TLRs and requisite adaptor molecules [33]. PAMP recognition specificity in vertebrates is determined by an N-terminal extracellular domain consisting of leucine-rich repeats (LRRs) [36]–[38]. Signal transduction takes place through a Toll/Interleukin 1 receptor (TIR) domain [29]–[34]. Recombination of these two principal domains of TLRs family has been hypnotized not to be random but follow specific rules dictate by the selective pressure of the environmental pathogens [39].

TLRs also do not act alone. Although all TLRs result in the activation of the transcription factor NF-κB and IFN regulatory factors (IRFs) [29]–[34], individual TLR signaling pathways differ in the type and nature of adaptor molecule, which are critical for activating downstream components of the signaling cascade. With the exception of TLR3, all TLRs make use of the adaptor molecule MyD88. TLR5, TLR7, TLR8, and TLR9 require no further adaptor molecules. Structurally MyD88 contains both a TIR domain and a Death domain. It is hypothesized that dimerization of the TLRs through ligand binding leads to a TIR-TIR interaction that recruits MyD88 through its TIR domain [32]. In vertebrates, MyD88 then activates IRAK4 via the interaction between the death domain of MyD88 and the death domain of IRAK4. IRAK4 in turn phosphorylates IRAK1. Phosphorylated IRAK1 then activates TRAF6 which undergoes ubiquitination leading to NF-κB activation. In invertebrates, MyD88 together with the adaptor Tube form a complex with the kinase Pelle, which in turn leads to the activation of Cactus, and ultimately to the activation of the NF-κB orthologs Dif and Dorsal [40]–[42]. In the case of both TLR2 and TLR4, a second adaptor molecule, TIRAP, containing a TIR domain, is required for signal transduction and appears to play a structural role in the TIR-TIR interaction of TLR2 or TLR4 with MyD88 [29]–[34].

A second, MyD88-independent, signaling pathway is used exclusively by TLR3 and in addition to the MyD88-dependent pathway by TLR4. TLR3 and TLR4 make use of this pathway through the recruitment of the adaptor molecule TRIF [32]. In the case of TLR4, an additional molecule, TRAM appears to be necessary for coupling the TIR domain of TLR4 to the TIR domain of TRIF in a manner analogous to TIRAP and MyD88. In addition to the TIR domain, TRIF contains an N-terminal binding domain for TRAF6 and the kinases IKKi and TBK-1 as well as a C-terminal binding domain for RIP-1. RIP-1 has been shown to be an essential mediator of NF-κB activation for both TLR3 and TLR4 [43]. Similarly, TBK and IKKi are required to activate IRF-3 [34].

Because adaptor molecules are not simply links between receptors and their effectors, but are coordinators of the signaling pathway dynamic, they are a critical factor shaping the evolution of the TLRs and their protein domains. In this study we report a molecular cophylogeny of the TLRs together with their signaling cognate adaptor molecule viewed through their shared TIR domain. We use these data to show how early gene duplications shaped extant TLR and adaptor associations, confirm the relationship between invertebrate and vertebrate TLR families, evince the ancient origin of the TIR domain and its associated adaptors, and suggest a model for the emergence of the MyD88 independent pathways. This represents a novel approach to the analysis of the TLR evolution through the phylogeny of the TIR signaling domain of related proteins in the signaling cascade.

## Results

### Phylogeny of TIR Domain: TLRs and Adaptor Molecules

**Figure 1:**
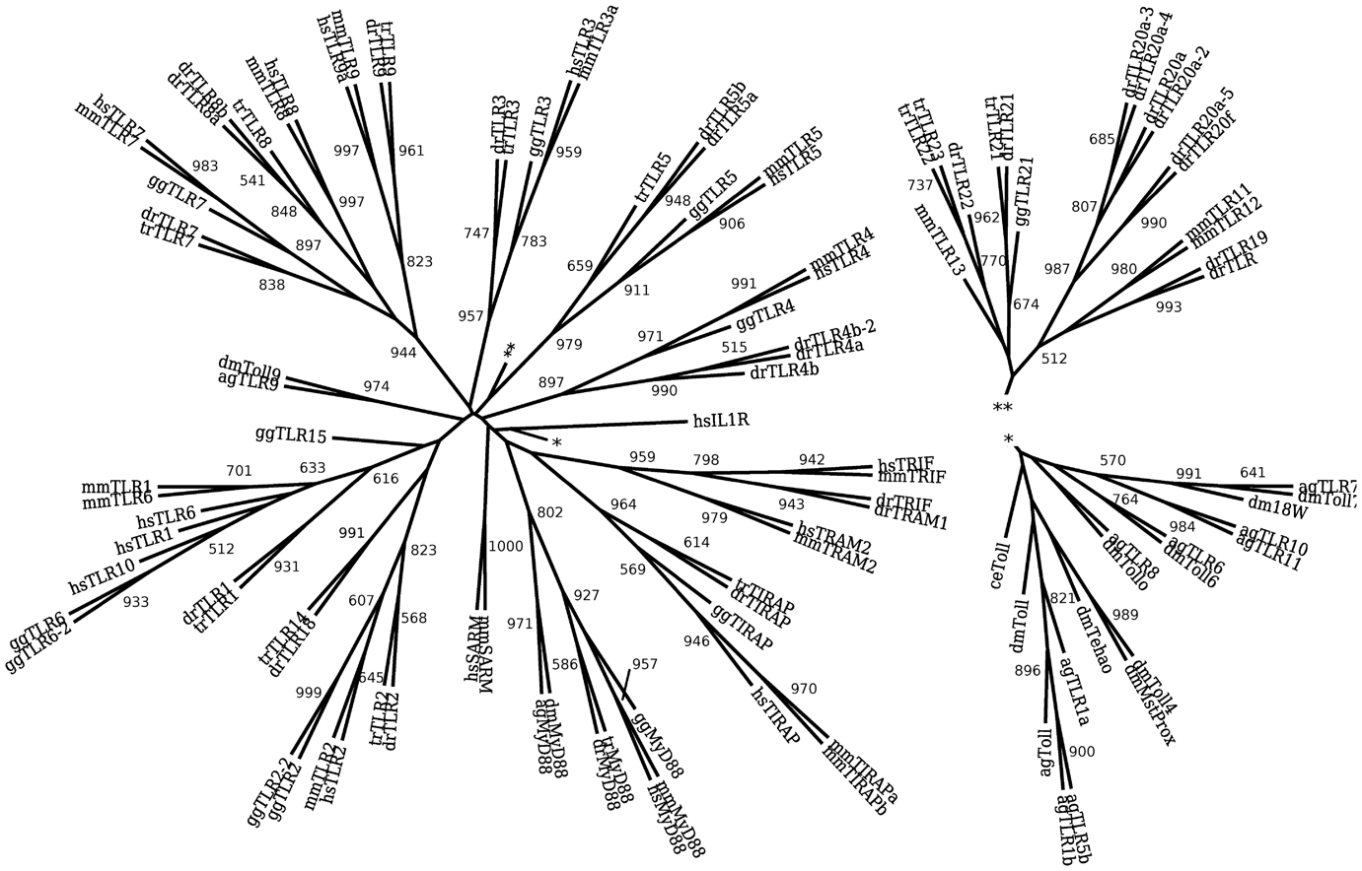
Maximum likelihood phylogeny of the TIR domain of the TLR family and TLR adaptor molecules shows strong support for individual branches corresponding to vertebrate TLR3, TLR4, TLR5, and invertebrate TLR9. Additionally, the invertebrate TLR9 taxa cluster with vertebrate TLRs outside of the main invertebrate TLR branch. A single cluster contains both vertebrate and invertebrate MyD88, but other TLR adaptor molecules form three groups: TIRAP, SARM; and a single branch for both TRIF and TRAM. The tree is rooted by the outgroup TIR domain Homo sapiens interleukin 1 receptor. The numbers indicate boot-strap support out of 1000. Only values above 500 are indicated.

Following the protocol described in methods section, we analyzed TIR domain of TLRs family members and their adaptors. The maximum likelihood (ML) phylogeny reconstructed from the TIR domains of the TLRs and TLR adaptor molecules is shown in figure 1. An essentially equivalent minimum evolution (ME) phylogeny is given in figure S1. Both reconstructions are calculated from the same multiple sequence alignment and both resulting trees are rooted by the TIR domain of the *Homo sapiens*interleukin-1 receptor. Consistent with previous phylogenies of the TIR domain of the TLRs, in both reconstructions, strong boot-strap support is observed for individual branches corresponding to vertebrate TLR3, TLR4, TLR5, and invertebrate TLR9. In addition a single cluster composed of vertebrate TLR7, TLR8, and TLR9 shows strong support. As observed by Kanzok *et. al*. [22], the invertebrate TLR9 taxa cluster with vertebrate TLRs outside of the main invertebrate TLR branch and are more closely associated with the vertebrate TLRs. Among TLR adaptor molecules, both reconstructions show strong boot-strap support for individual clusters for TIRAP and SARM; and a single branch for both TRIF and TRAM. Moderate boot-strap support is observed in both reconstructions for a single cluster containing both vertebrate and invertebrate MyD88. Neither reconstruction supports a common ancestor for the TIR domains occurring in the TLR family and the TIR domains occurring in the adaptor molecules.

The phylogenies of the TIR domain of the TLRs not including the adaptor molecules and the phylogenies of the full length TLRs show receptor groupings consistent with the groupings found in the phylogeny of the TIR domain of the TLRs calculated with the adaptor molecules. This suggests that the adaptor molecules have co-speciated with the TLRs. ML and ME reconstructions of the TIR domain of the TLRs are shown in figure S2 (ML) and figure S3 (ME). Both reconstructions are rooted with respect to an outgroup containing the TIR domain of *Homo sapiens*MyD88. Again individual clusters for vertebrate TLR3, TLR4, TLR5, and invertebrate TLR9 are shown to be strongly supported in both reconstructions as is a strongly supported branch containing vertebrate TLR7, TLR8, and TLR9. Placement and support of the remaining vertebrate TLRs with no human ortholog are essentially identical to the reconstruction including the TLR adaptor molecules. Boot-strap values observed in the focused reconstruction are somewhat larger than the boot-strap values observed in the reconstruction including adaptor molecules. However, as observed previously, boot-strap supports observed in the ML reconstruction remain uniformly lower than the ME reconstruction.

ML and ME reconstructions of the full-length TLRs rooted by the outgroup consisting of the protein coded by the *Nicotiana glutinosa* gene of the full-length TLR are displayed in figures S4 and S5 respectively. Consistent with the TIR domain phylogenies reconstructed with and without TLR adaptor molecules, vertebrate TLR3, TLR4, and TLR5 form strongly supported individual branches and a single strongly supported cluster contains vertebrate TLR7, TLR8, and TLR9. As observed in all phylogenies restricted to the TIR domain, within the TLR7, TLR8, and TLR9 cluster, TLR9 is observed to associate more distantly than TLR7 and TLR8. Similarly both ME and ML have a strongly supported individual cluster for TLR2 and a strongly supported cluster for TLR1, TLR6, and TLR10 as observed in the TIR restricted reconstructions.

**Figure 2:**
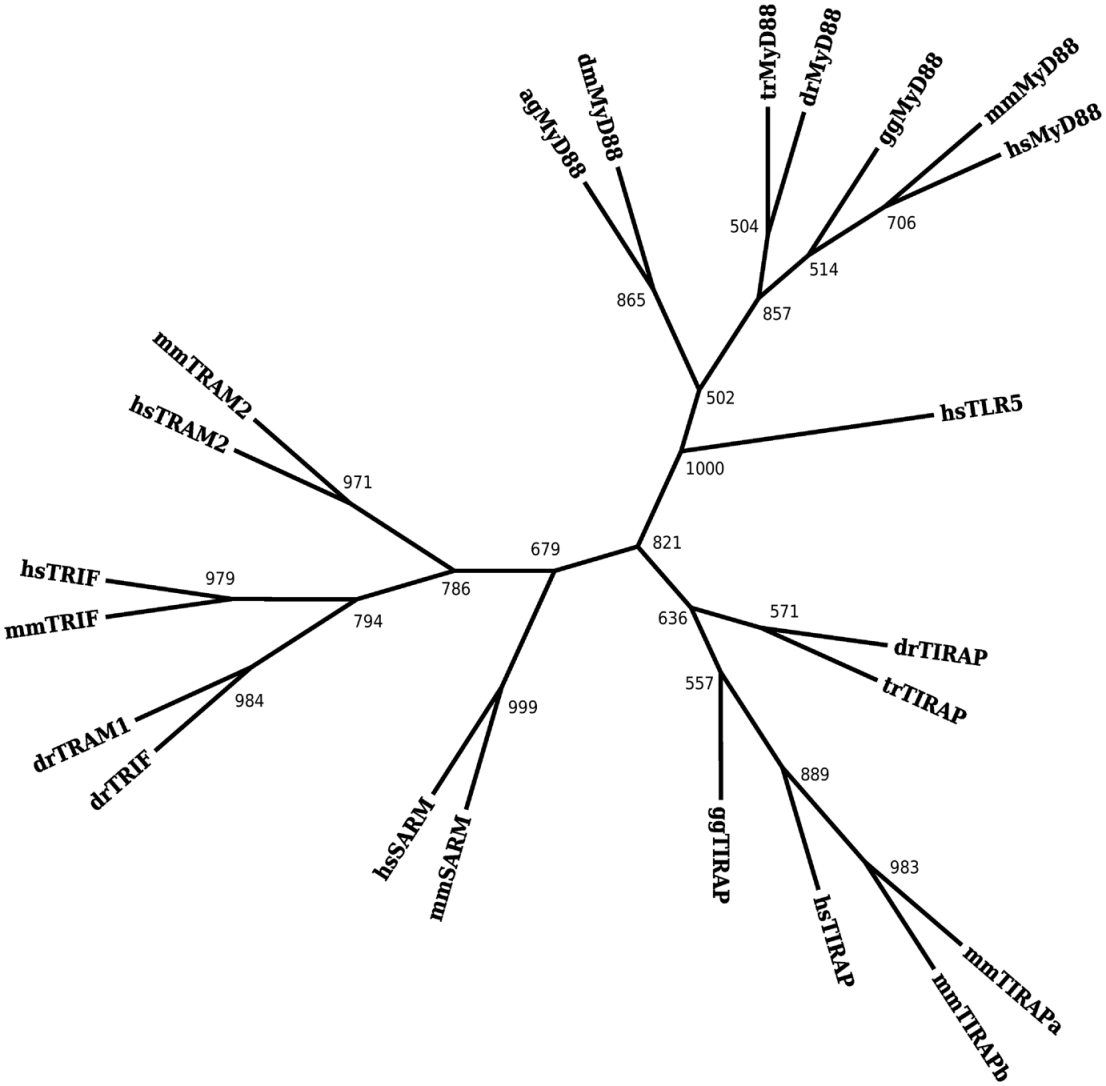
Maximum likelihood phylogeny of the TIR domain of the TLR adaptor molecules alone is consistent with the topology observed in **Figure 1**. The tree is rooted by the outgroup TIR domain of Homo sapiens TLR5. The numbers indicate boot-strap support out of 1000. Only values above 500 are indicated.

Reconstructions of the phylogeny of the TIR domain of the adaptor molecules along are shown in figures 2 (ML) and S6 (ME). Both reconstructions are rooted by the TIR domain of *Homo sapiens*TLR5. Three branches are moderately supported in both reconstructions: a MyD88 branch including both invertebrate and vertebrate MyD88, a branch containing TIRAP, and a branch composed of TRAM, TRIF, and SARM. The branching of MyD88 from the remaining adaptor molecules is well-supported and there is modest support in both reconstructions for a common ancestor to invertebrate and vertebrate MyD88 as observed in the reconstructed phylogeny of TIR including the TLRs.

Within the TRAM, TRIF and SARM branch, there is moderate to strong support in both reconstructions for a common ancestor to the MyD88-independent pathway adaptors: TRAM and TRIF. SARM also appears to be associated to this branch although it is observed with less support, consistent with the hypothesis that SARM is a late addition to the adaptors family [44]. The association of TRAM and TRIF was observed in both reconstructions of the TIR phylogeny including the TLRs; however, the association with SARM to this grouping was unresolved when the TLRs were included. These data suggest that the MyD88-independent pathway arose once then diversified–much like other TLRs–through a series of gene duplications.

### Cophylogeny of TIR Domain: TLRs and Adaptor Molecules

**Figure 3:**
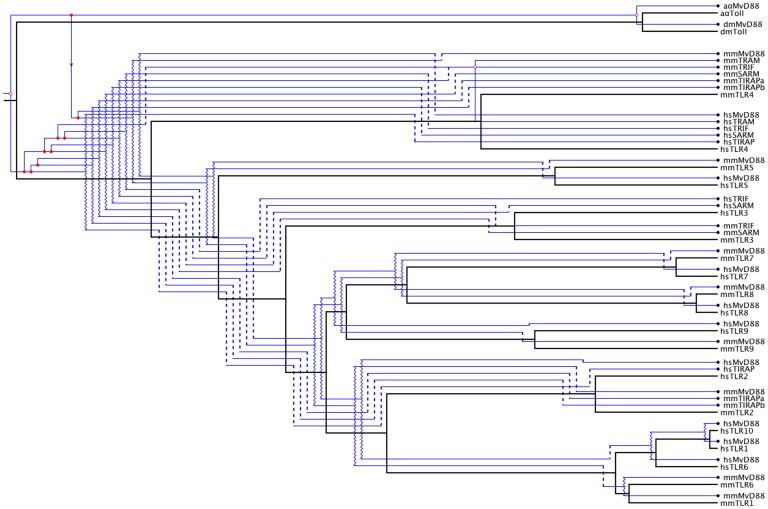
Embedding of the phylogeny of the TIR domain of the adaptor molecules into the phylogeny of the TIR domain of the TLRs for mouse and human shows that early gene duplication gave rise to extant adaptor families and paralleled the diversification of TLRs. Open circles indicate cospeciation events. Closed circles indicate duplication events. Wavy lines indicate failure to diverge and dashed lines indicate loss. Lines with arrows denote "host" switching- the co-option of an adaptor by a different TLR. These data are consistent with the vertebrate MyD88 begin recruited from an earlier invertebrate-like MyD88 that was likely associated with TLR9-like TLRs.

The data above suggest a long standing evolutionary relationship between TLRs and their adaptor molecules. To uncover the dynamics of this relationship, the TIR domain of the adaptor molecule phylogeny was embedded into the phylogeny of the TIR domain of the TLRs restricted to human and mouse (Figure 3). All generated minimal cost embeddings share essentially the same topology and have the same cost. Generation of 1000 random cophylogenies indicate that these embeddings are significant at the 1.2% level.

These embeddings suggest an early cospeciation of the MyD88 adaptor molecule branch from the branch containing ancestors of the remaining adaptors with the MyD88 ancestor associated to the invertebrate TLR branch and the common ancestor of TIRAP, TRIF, TRAM, and SARM associated with the vertebrate TLRs. In the invertebrate TLR branch, a later cospeciation is observed with the divergence of individual invertebrate TLRs. Vertebrate MyD88 is indicated as resulting from an early host switch from the invertebrate MyD88 branch. This branch then duplicates ultimately resulting in distinct human and mouse MyD88.

The adaptor molecules TIRAP, TRIF, and SARM are indicated as resulting from early duplication events associated with the vertebrate TLR phylogeny. Ancestors of the molecules specialize at a later stage into extant forms for mouse and human. A distinct pattern of cospeciation is observed with TRAM where adaptor molecule phylogeny branches for mouse and human TRAM diverge roughly contemporaneously with the divergence of TLR4 for mouse and human, the ancestor of TRAM being the result of a duplication event within the TIRAP, TRIF, and SARM.

## Discussion

The need to conserve the capability to recognize specific pathogen signature while at the same time allowing for the development of new platforms on which to build more complex signaling networks drives the evolution of TLR signaling. The high degree of conservation of recognized ligands between orthologous TLRs can be, in part, explained by the observation that TLRs have evolved to recognize microbial PAMPs that cannot be easily and viable mutated in pathogens. Nevertheless pressure to maintain endogenous signaling networks remains a significant constraint. The difficulty in satisfying these competing requirements suggests the importance of adaptors molecules. Phylogenetic analysis of TLRs and their adaptor molecules can reveal the history and dynamic of these competing requirements on the evolution of TLRs.

The inclusion of the transmembrane and extracellular domains of the receptor in the analysis shows how dominant these specific functional domains are dictating the TLR phylogeny. Comparing the reconstructions of the full-length TLRs with the TLR family phylogeny restricted to the TIR signaling domain shows that the large extracellular domain drives the topology of the phylogeny. This observation suggests that functional constraints in PAMP recognition dictate the evolution of this interface. All reconstructions support an interpretation of the clustering based on pattern recognition ligand [16]–[22]. Branches of both reconstructions correspond to various TLR PAMPs. Two branches correspond to TLRs recognizing nucleic acids: TLR3 recognizing double stranded RNA; and TLR7, TLR8 and TLR9 recognizing single-stranded RNA and viral CpG. The remaining branches correspond to membrane components: a single branch for TLR4, recognizing lipopolysacharide; a single branch for TLR5, recognizing flagellin; and one or more branches for TLR1, TLR2, TLR6, and TLR10 recognizing bacterial lipopeptide ligands.

In contrast, restriction of the phylogenetic analyses to the TIR domain highlights the evolution of TLR signaling. Phylogenetic reconstructions focusing on the TLRs and on their adaptor molecules separately result in essentially identical configurations suggesting that the need to maintain a particular suite of protein-protein interfaces between the TIR domain and the adaptor molecules is applying an evolutionary constraint. Among TLRs all reconstructions show strong support for clusters consisting of TLR3, TLR4, and TLR5 individually, and a single cluster for TLR7, TLR8, and TLR9. Moderate support is found for two clusters consisting of TLR2 and TLR1, TLR6, and TLR10. In most reconstructions these two clusters are joined into a single modest to moderately well-supported cluster.

The phylogenetic analyses of the TIR domain of the TLRs together with their adaptor molecules support the hypothesis that adaptor molecule usages and signaling pathway are ancestral characteristics of the TLRs. Among the TLRs that use MyD88 exclusively, well-supported branches for TLR7, TLR8, and TLR9 and for TLR5 are observed. Similarly, TLR3, using TRIF exclusively, and TLR4 using MyD88, TIRAP, TRIF, and TRAM, form well-supported, individual branches as do the adaptor molecules used by the MyD88-independent pathway: TRIF and TRAM. The hypothesis is supported to a lesser extent by the TLRs using both MyD88 and TIRAP: TLR2 and TLR1, TLR6, and TLR10; with TLR2 and TLR1, TLR6, and TLR10, belonging moderately supported branches.

Comparing to the TIR domain reconstructions including the TLR TIR domains as well as the TLR adaptor molecule TIR domains, the clustering of TRIF and TRAM is strongly supported over all reconstructions. Similarly all reconstructions support invertebrate and vertebrate MyD88 branches. Furthermore when compared in the context of TLR TIR domains, reconstructions strongly support a common ancestor for these two MyD88 branches suggesting that the evolution of MyD88 adaptor-TIR domain is ancient. Additionally, all reconstructions show at least moderate support for an individual TIRAP branch. These data shed additional light on recent results concerning the coevolution of TRIF, TRAM, and TIRAP with the respective TLR3/22 and TLR2 signaling pathway [40].

The hypothesis that adaptor molecule usage is an ancestral characteristic of the Toll receptor signaling is further supported by the reconstructions of the adaptor molecules phylogeny. Well-supported monophyletic clusters are observed for vertebrate MyD88, invertebrate MyD88, and both of the adaptor molecules required in the MyD88-independent pathway: TRIF and TRAM. The adaptor molecule TIRAP, used by TLR1, TLR2, TLR4, TLR6, and TLR10, is isolated to a single cluster in all reconstructions. There is no observed evidence for a common ancestor between the TIR domains of any of the TLRs and the TIR domains of any of the adaptor molecules. This pattern suggests that the TIR domains of adaptor molecules are not derived from the TIR domains of TLRs and that the primordial TLR may have co-opted an existing adaptor based signaling system.

The cophylogeny of the TLRs with their adaptor molecules reveal how adaptor molecule parallels TLR evolution, pinpoints key gene duplication events (Figure 3), and in general highlights how Toll receptor signaling constrains the evolution of this gene family. The embedding of the adaptor molecule phylogeny into the TLR phylogeny supports an early cospeciation with the divergence of invertebrate and vertebrate TLRs. Interestingly, the MyD88 pathway is shown to result from a duplication and subsequent host switch of the MyD88 ancestor from the invertebrate TLR branch to the vertebrate TLR branch. In the case of vertebrate TLR2 and TLR4, adaptor molecules from both branches of the initial vertebrate and invertebrate cospeciation are required. In particular, the adaptor molecule TIRAP associates to the vertebrate TLR branch of the cophylogeny. In contrast, the MyD88-independent pathway for TLR3 and TLR4, using TRIF and TRAM, derives completely from the adaptor molecules branch associated to the vertebrate TLRs.

The outcome of pathogen recognition is critically dependent on the TLR-restricted utilization of TIR domain-containing adaptor molecules to drive stimulus specific responses. The phylogenetic analysis reported here indicates an independent evolutionary origin for the MyD88-dependent and MyD88-independent pathways. Furthermore this study indicates a common ancestor for vertebrate and invertebrate MyD88, therefore suggesting a very ancient origin of the MyD88-dependent pathway. Through the phylogeny of TIR domain, this work points out the evolution of a complex signaling network by the different usage of adaptor molecules, otherwise lost in the full length sequences analysis. Overall, this confirms a recently study in human, which reports a different form of selection between TLRs family members and their adaptors, and showes that the adaptors represent a more essential and no redundant component of the TLRs signaling cascade [45]. Moreover, phylogenetic analysis of domain of receptor and adaptor molecules proves to be a useful conceptual framework providing context to diverse empirical results, suggesting hypotheses on pathogens and cellular recognition machinery to be tested both computationally and experimentally.

## Materials and Methods

An initial set of amino acid sequences for the TLRs was obtained from NCBI GenBank [46] through a combination of extensive keyword searches and BLAST searches. Partial sequences were removed and a limited number of sequences noted in earlier communications [19]–[21] were included. Redundant sequences were removed through all-by-all pairwise sequence alignment by CLUSTALW 2.0.10 [47]. The complete list of amino acid sequences used is available in the supplemental materials.

The resulting set of amino acid sequences was submitted to multiple sequence alignment by ProbCons 1.12 [47]. From the resulting multiple sequence alignment a hidden Markov model (HMM) was constructed by HMMER 2.3.2 [48] for the full-length TLR. The NCBI GenBank reference sequence database [49] for the organisms: *Anopheles gambiae*, *Caenorhabditis elegans, Drosophila melanogaster, Danio rerio, Takifugu rubripes, Gallus gallus, Mus musculus, Homo sapiens*, was queried for the matches to the full-length HMM model resulting in the final set of full-length amino acid sequences submitted to phylogenetic analysis. These species have been chosen to provide the most phylogenetic insight into TLR signaling, in general, and the interactions of the TLRs with their adaptor molecules, in particular, across a wide spectrum of organisms. [50]–[52].

The TIR domain was predicted from the initial set of amino acid sequences using the appropriate Pfam [53] HMM models. For each domain a ProbCons multiple sequence alignment was constructed and from this alignment a TIR domain specific HMM model was specified. This TIR domain specific HMM model was then used to search the NCBI GenBank reference sequence database for the organisms under consideration. Sequence matches to the domain specific HMM were restricted to the subsequence predicted by the model. A second set of amino acid sequences corresponding to the TIR domain of adaptor molecules was constructed analogous to the sequence set for the TIR domains of the TLRs. These two sets were combined and the resulting set of TIR domain specific amino acid sequences was submitted to phylogenetic analysis.

Multiple sequence alignments constructed by ProbCons were submitted to both maximum likelihood and minimum evolution phylogeny reconstruction. Maximum likelihood reconstructions were calculated using *proml* in the PHYLIP 3.68 package [54]. Distance matrices for the minimum evolution reconstruction were calculated with *protdist* found in PHYLIP. Minimum evolution phylogeny was constructed from this distance matrix by *fastme* [55]. In both cases, 1000 boot-strap replications were generated using *seqboot* and the consensus phylogeny was assembled with *consense*, both from the PHYLIP package. FigTree 1.2.1 (http://tree.bio.ed.ac.uk/software/figtree/) was used for both initial visualization and final production of figures of the resulting phylogenetic trees.

Cophylogenetic embeddings of the TIR domain of the adaptor molecules into the phylogeny of the TIR domain of the TLRs together with empirical estimates of significance were constructed with Jane [56]. Thirty minimum cost trees were constructed based on default event costs. Statistical significance of the embedding was calculated with respect to a sample size of 1000.

## Supporting Information

Figure S1 **Minimum evolution phylogeny of the TIR domain of the TLR family and TLR adaptor molecules.** The tree is rooted by the outgroup TIR domain *Homo sapiens*interleukin 1 receptor. The numbers indicate boot-strap support out of 1000. Only value above 500 are indicated.
(TIFF)

Figure S2 **Maximum likelihood phylogeny of the TIR domain of the TLR family alone.** The tree is rooted by the outgroup TIR domain *Homo sapiens*MyD88. The numbers indicate boot-strap support out of 1000. Only value above 500 are indicated.
(TIFF)

Figure S3 **Minimum evolution phylogeny of the TIR domain of the TLR family alone.** The tree is rooted by the outgroup TIR domain *Homo sapiens*MyD88. The numbers indicate boot-strap support out of 1000. Only value above 500 are indicated.
(TIFF)

Figure S4 **Maximum likelihood phylogeny of the TLR family reconstructed from the complete amino acid sequence.** The tree is rooted by the outgroup *Nicotiana glutinosa*N. The numbers indicate boot-strap support out of 1000. Only value above 500 are indicated.
(TIFF)

Figure S5 **Minimum evolution phylogeny of the TLR family reconstructed from the complete amino acid sequence.** The tree is rooted by the outgroup *Nicotiana glutinosa*N. The numbers indicate boot-strap support out of 1000. Only value above 500 are indicated.
(TIFF)

Figure S6 **Minimum evolution phylogeny of the TIR domain of the TLR adaptor molecules alone.** The tree is rooted by the outgroup TIR domain *Homo sapiens*TLR5. The numbers indicate boot-strap support out of 1000. Only value above 500 are indicated.
(TIFF)

Supplementary Materials and Methods S1 (DOCX)
