# Supplementary figures and images for: Phylogeny of Toll-Like Receptor Signaling: Adapting the Innate Response
Source: PLoS One. 2013 Jan 11;8(1):e54156. doi: 10.1371/journal.pone.0054156 (PMC3543326; doi:10.1371/journal.pone.0054156)

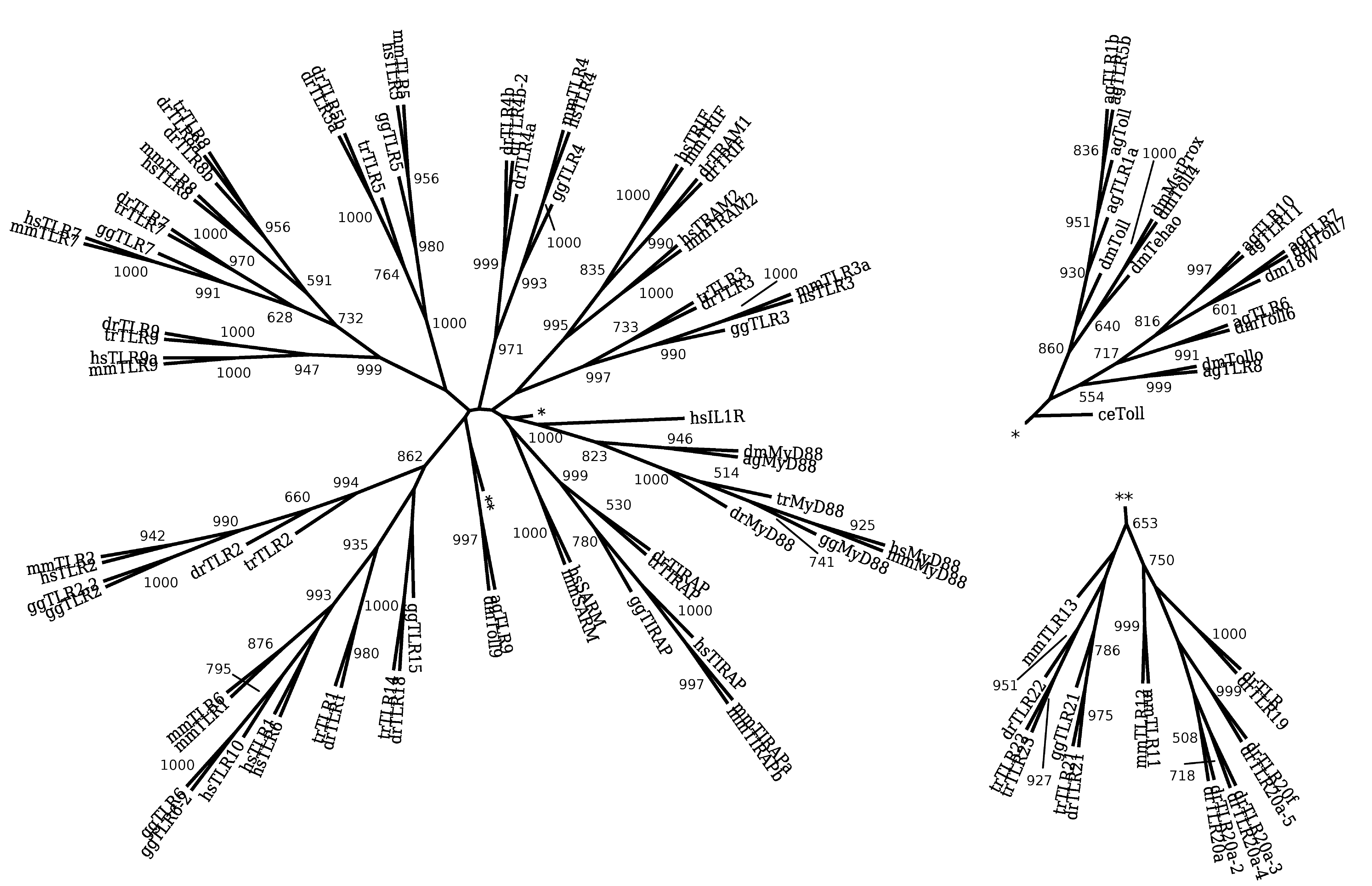

Supplement: Figure S1 — Minimum evolution phylogeny of the TIR domain of the TLR family and TLR adaptor molecules. The tree is rooted by the outgroup TIR domain Homo sapiensinterleukin 1 receptor. The numbers indicate boot-strap support out of 1000. Only value above 500 are indicated. (TIFF) [file pone.0054156.s001.tiff]

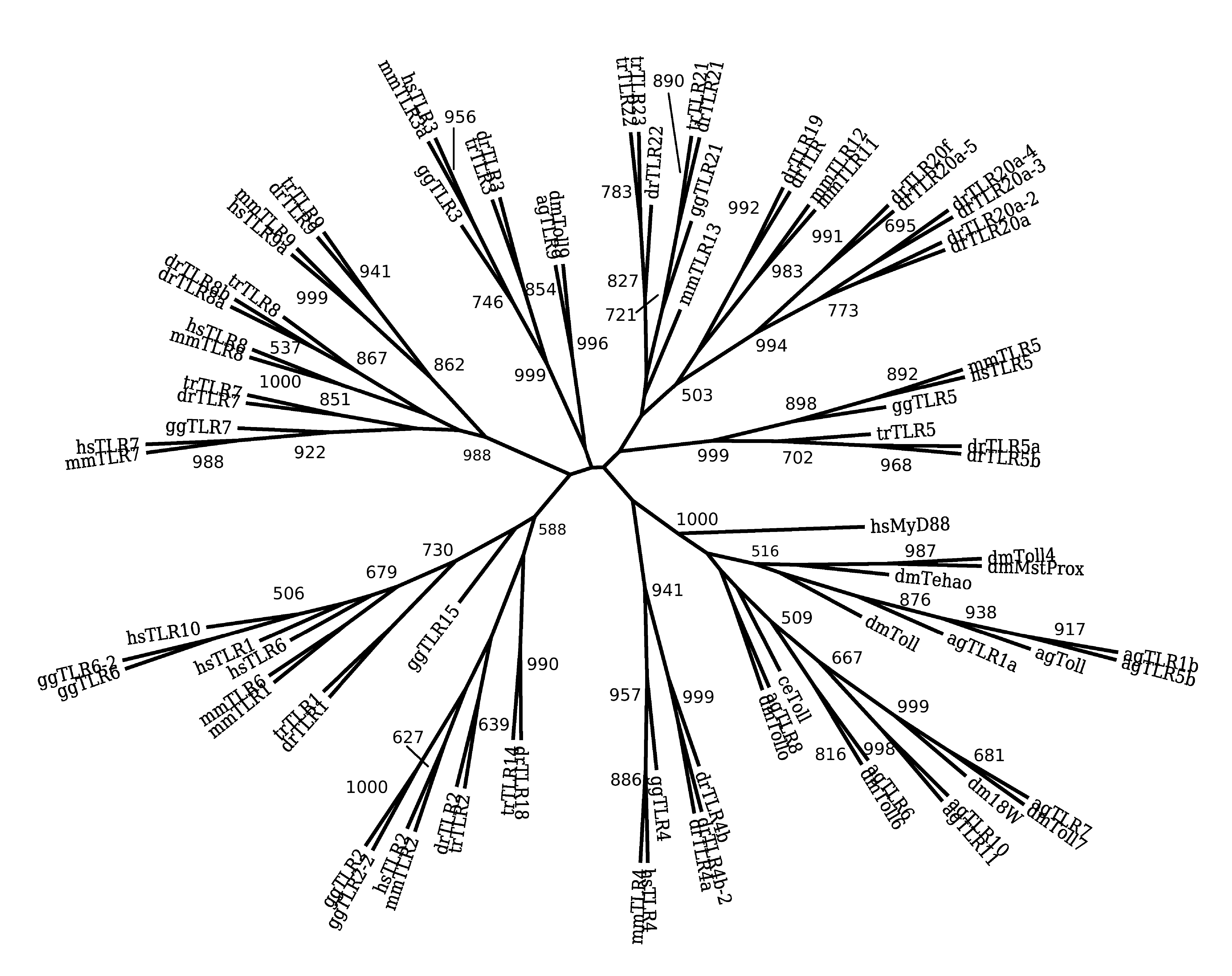

Supplement: Figure S2 — Maximum likelihood phylogeny of the TIR domain of the TLR family alone. The tree is rooted by the outgroup TIR domain Homo sapiensMyD88. The numbers indicate boot-strap support out of 1000. Only value above 500 are indicated. (TIFF) [file pone.0054156.s002.tiff]

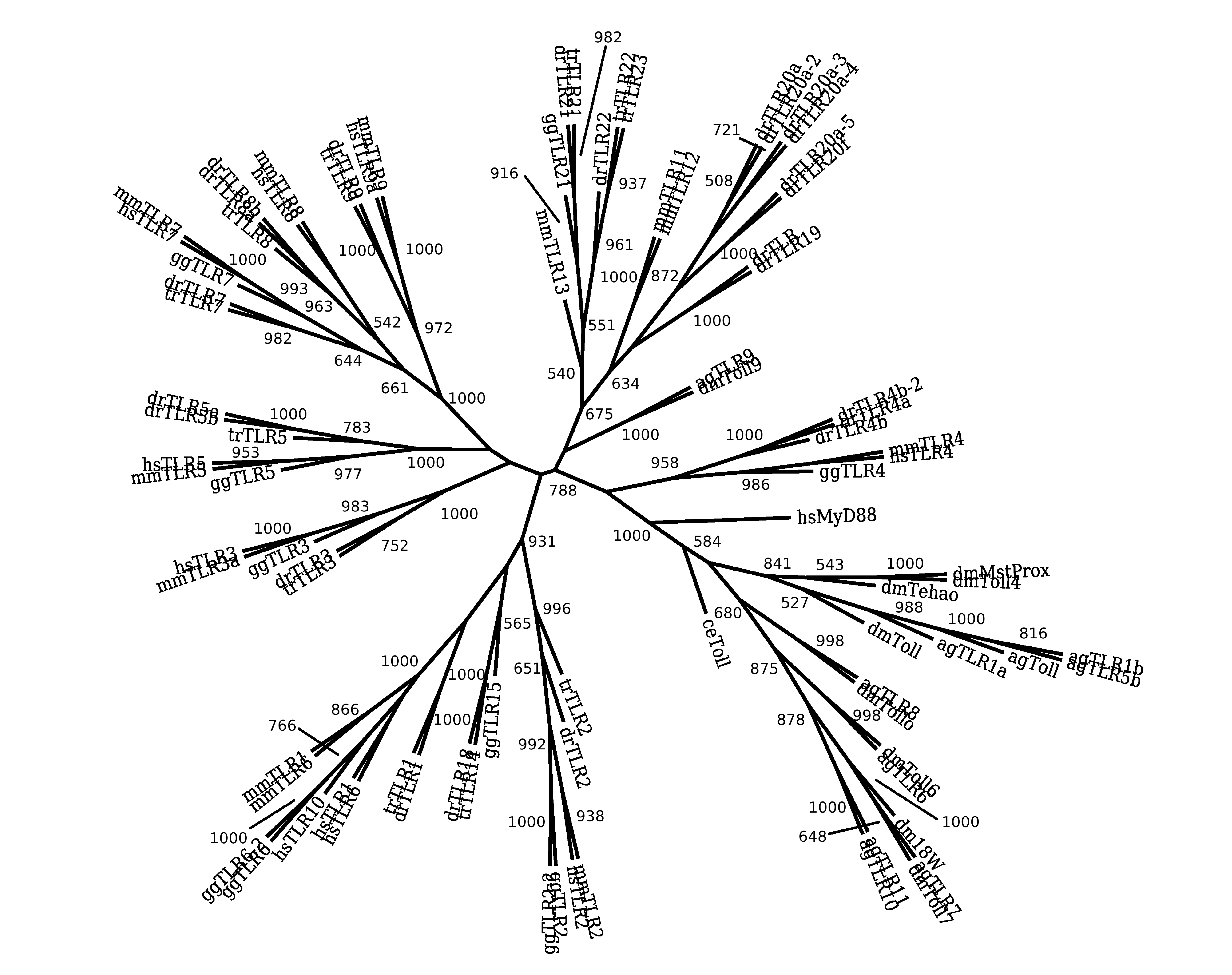

Supplement: Figure S3 — Minimum evolution phylogeny of the TIR domain of the TLR family alone. The tree is rooted by the outgroup TIR domain Homo sapiensMyD88. The numbers indicate boot-strap support out of 1000. Only value above 500 are indicated. (TIFF) [file pone.0054156.s003.tiff]

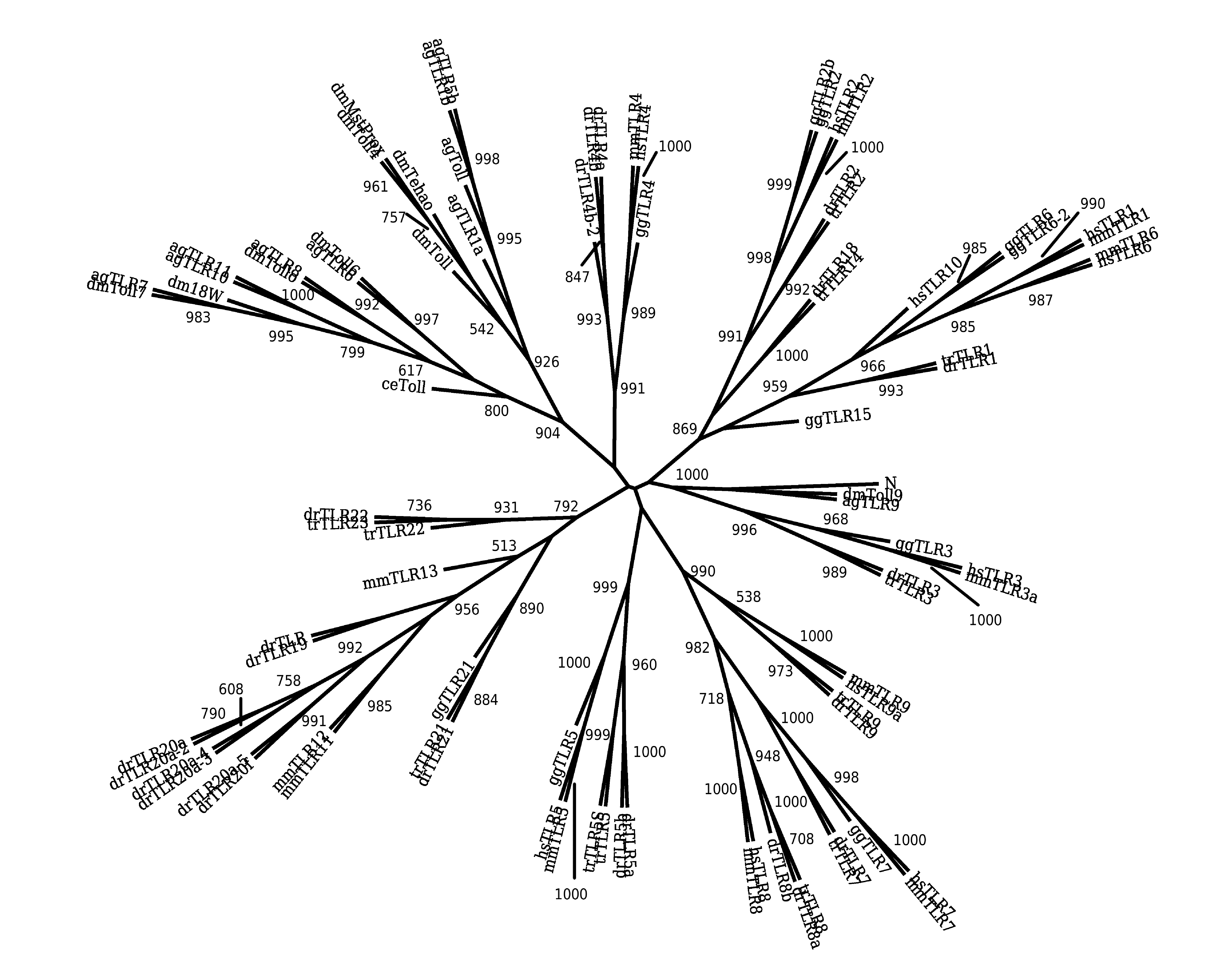

Supplement: Figure S4 — Maximum likelihood phylogeny of the TLR family reconstructed from the complete amino acid sequence. The tree is rooted by the outgroup Nicotiana glutinosaN. The numbers indicate boot-strap support out of 1000. Only value above 500 are indicated. (TIFF) [file pone.0054156.s004.tiff]

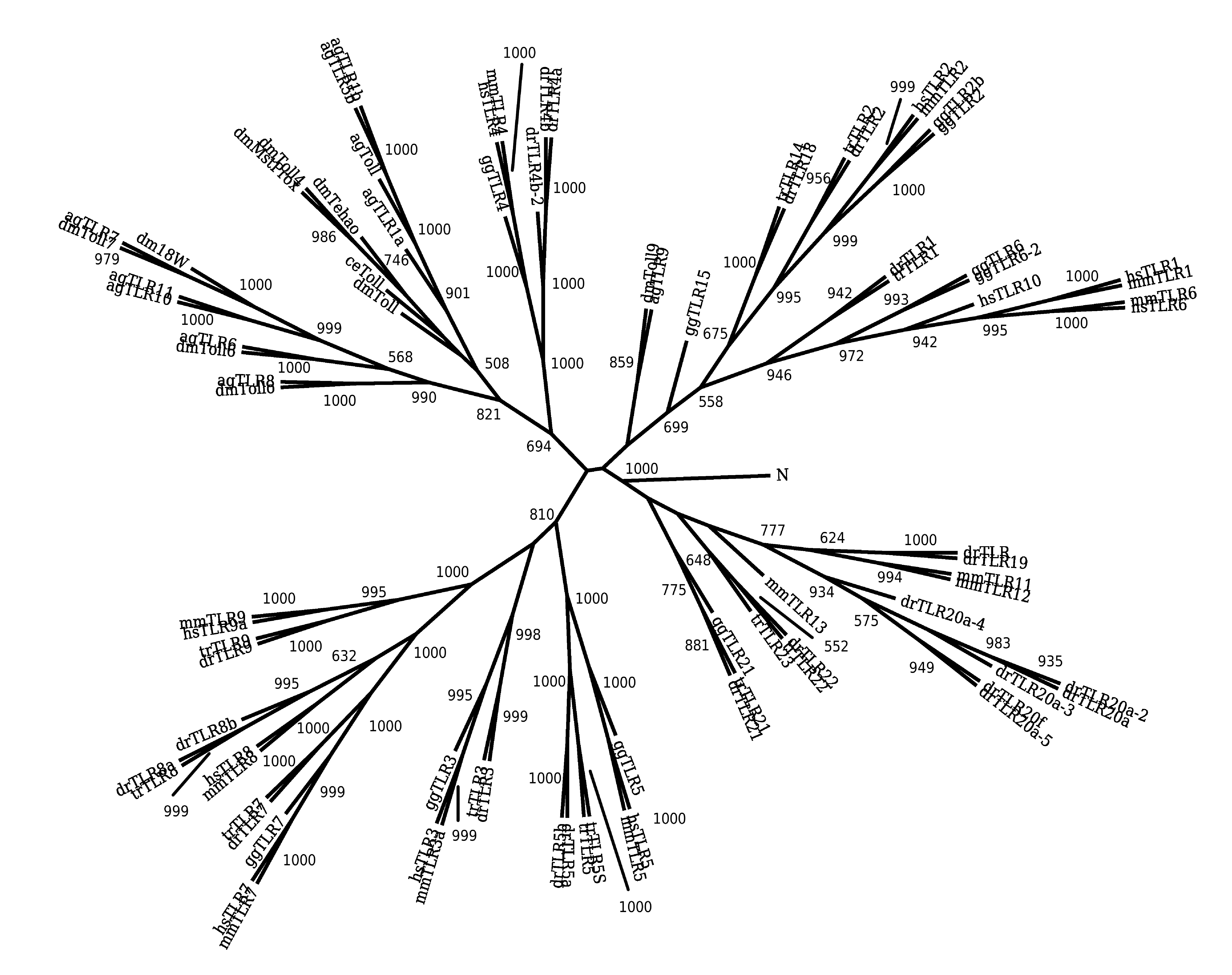

Supplement: Figure S5 — Minimum evolution phylogeny of the TLR family reconstructed from the complete amino acid sequence. The tree is rooted by the outgroup Nicotiana glutinosaN. The numbers indicate boot-strap support out of 1000. Only value above 500 are indicated. (TIFF) [file pone.0054156.s005.tiff]

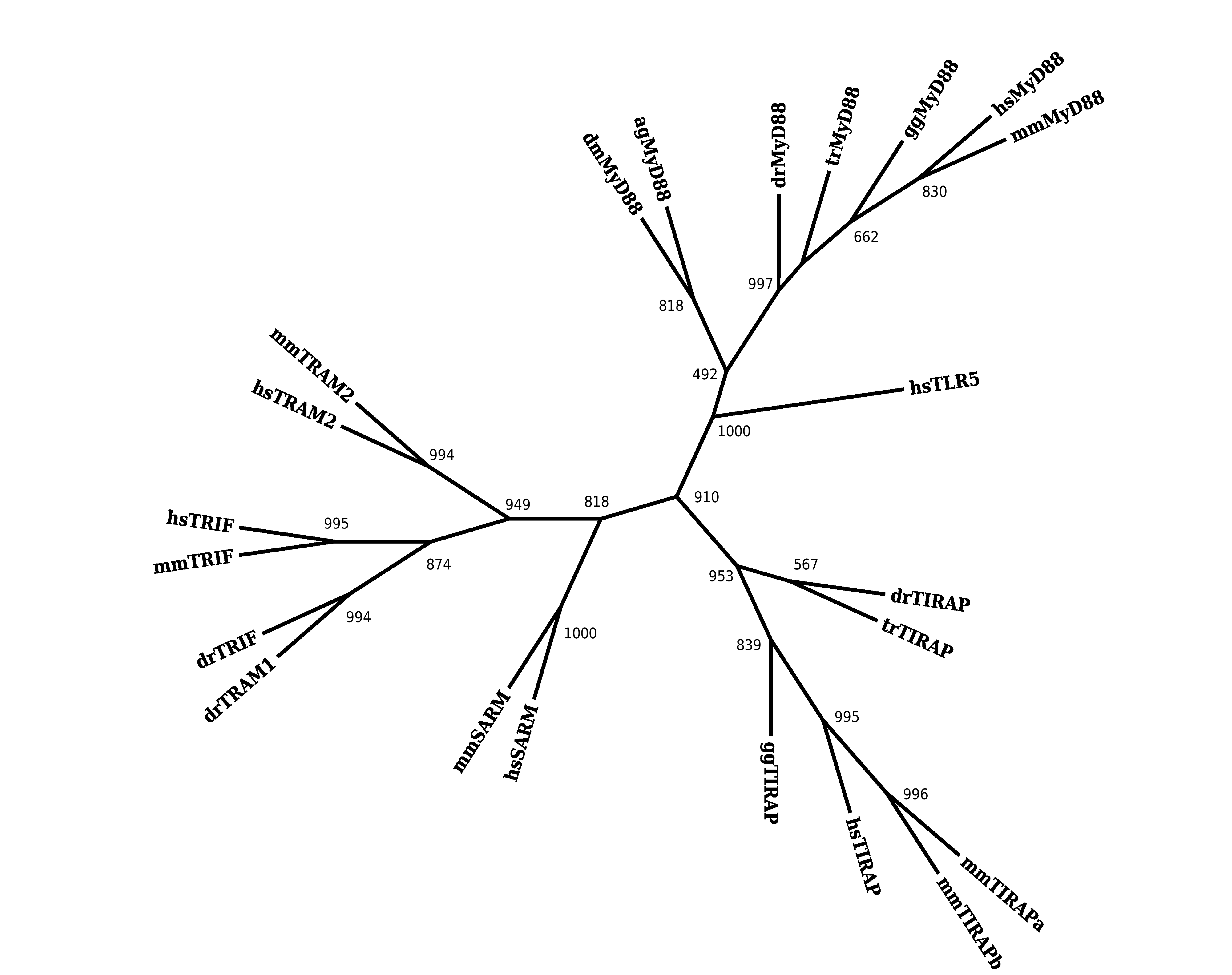

Supplement: Figure S6 — Minimum evolution phylogeny of the TIR domain of the TLR adaptor molecules alone. The tree is rooted by the outgroup TIR domain Homo sapiensTLR5. The numbers indicate boot-strap support out of 1000. Only value above 500 are indicated. (TIFF) [file pone.0054156.s006.tiff]
