# Supplementary material for: Phylogeny of Toll-Like Receptor Signaling: Adapting the Innate Response
Source: PLoS One. 2013 Jan 11;8(1):e54156. doi: 10.1371/journal.pone.0054156 (PMC3543326; doi:10.1371/journal.pone.0054156)
Supplement: Supplementary Materials and Methods S1 — (DOCX) [file pone.0054156.s007.docx]

**Supplemental Materials**

TIR domain with adoptor molecules

The differences between the ML and ME reconstructions of the TIR domains of the TLRs and their adoptor molecules are essentially differences in boot-strap support. Broadly speaking the ML reconstruction breaks moderately and modestly supported clusters in the ME reconstruction in to several smaller clusters in the ML reconstruction. This is indeed the case with vertebrate TLR1, TLR2, TLR6, TLR10, *Takifugu rubripes*TLR14, *Gallus gallus*TLR15, and *Danio rerio*TLR18; all of the remaining vertebrate TLRs for which there exist no *Homo sapiens*orthologue, *e.g.*murine TLR11, TLR12, and TLR13; as well as the invertebrate TLRs with the exception of invertebrate TLR9. The ME reconstruction shows a moderately supported branch for TLR1, TLR2, TLR6, and TLR10, including *Takifugu rubripes*TLR14, *Gallus gallus*TLR15, and *Danio rerio*TLR18; whereas the ML reconstruction includes a strongly supported branch for TLR14 and TLR18, a moderately supported branch for TLR2, and a modestly supported branch for TLR1, TLR6, and TLR10. Similarly the modestly supported cluster in the ME reconstruction containing all the remaining vertebrate TLRs with no *Homo sapiens*ortholog fractures in the ML reconstruction into a modestly supported individual cluster for TLR21; a modestly supported cluster for TLR11, TLR12, TLR19, and TLR20; and a moderately supported cluster for both TLR22 and TLR23. Likewise, two modestly supported invertebrate TLR clusters in the ME reconstruction are reduced to several clusters in the ML reconstruction: one modestly supported cluster contains *Anopheles gambiae* , TLR10, and TLR11 and *Drosophila melanogaster* Wheeler and Toll7; one moderately supported branch corresponds to invertebrate TLR6; a strongly supported branch for *Drosophila melanogaster* and MstProx; and a single cluster of moderate support for *Anopheles gambiae* a, TLR1b, TLR5b, and Toll.

TIR domain alone

Differences between the ME and ML reconstructions of the TIR domain of the TLRs alone are again limited to differences in boot-strap support; however, in the focused reconstruction, some of these differences are substantial. In particular, the ME branch containing TLR1, TLR2, TLR6, and TLR10, as well as TLR14, TLR15, and TLR18 shows strong boot-strap support measured at 931/1000 whereas the same cluster in the ML reconstruction is rated at only 588/1000.

The reconstruction of the TLR TIR domain among the remaining invertebrate TLRs differs somewhat in the focused reconstruction from the reconstruction including the adoptor molecules. A single modestly supported branch containing all the remaining invertebrate TLRs, including the single Toll receptor found in *Caenorhabditis elegans,* observed in the ME reconstruction. This cluster splits into two modestly supported branches in the ML reconstruction: one branch containing *Anopheles gambiae* , TLR7, TLR10, and TLR11 and *Drosophila melanogaster* Wheeler, Toll6, and Toll7; and a second branch containing *Anopheles gambiae* a, TLR1b, Toll, and TLR5b and *Drosophila melanogaster* , Toll4, MstProx, and Tehao.

Full-length Receptor

Although both ML and ME reconstructions of the full length receptors show moderate support for including TLR1, TLR2, TLR6, and TLR10, with TLR14, TLR15, and TLR18 in a single branch, the association of the TLR2 cluster with the TLR1, TLR6, and TLR10 cluster differs somewhat in boot-strap support between the two reconstructions and depends on the placement of *Gallus gallus*TLR15. In the ME reconstruction the support for a single branch is much lower than observed in the ME reconstructions restricted to the TIR domain suggesting that significant differences between TLR2 and TLR1, TLR6, and TLR10 exist outside of the signaling domain. Interestingly, this observation is precisely the opposite of what is observed in the ML reconstruction. In the ML reconstruction boot-strap support for a single branch in much higher in the full-length reconstruction that in any of the reconstructions restricted to the signaling domain alone, suggesting similarities in the extracellular domain that are not found in the signaling domain.

The ME reconstruction shows moderate support for groups TLR3, TLR5, and TLR7, TLR8, and TLR9 into a single branch. This configuration is observed, although lacking in sufficient boot-strap support, in the ME reconstruction of the TLR TIR domains without TLR adoptor molecules. With the addition of TLR adoptor molecules, a weakly supported association of TLR5 with TLR7, TLR8, and TLR9 is observed. This latter configuration is observed without support in the ML reconstruction of the full-length receptors, but not in the reconstructions restricted to the TIR domain.

Among the remaining vertebrate TLRs that have no human ortholog, the reconstructions disagree on the strength of a single branch. The ML reconstruction shows a moderately supported branch containing all remaining vertebrate TLRs that posses no human ortholog. The ME reconstruction breaks this cluster into three moderately supported branches: *Mus musculus* and TLR12, *Danio rerio* , TLR19, and TLR20; TLR21 for *Danio rerio*, *Takifugu rubripes*, and *Gallus gallus*, and TLR22 for *Danio rerio*and *Takifugu rubripes,* TLR23 for *Takifugu rubripes.* Sub-branches within the single branch observed in the ML reconstruction are essentially similar to the ME grouping. This configuration is essentially consistent with previously observed reconstructions restricted to the TIR domain. The only significant differences lie in the placement and support of *Danio rerio*TLR and TLR19 within a larger cluster containing *Mus musculus*TLR11 and TLR12 and *Danio rerio*TLR20.

As observed in the reconstructions restricted to the TIR domain, in both ML and ME reconstructions, the invertebrate TLR9 receptors cluster outside of the main invertebrate branch. Both reconstructions also essentially concur on the clustering of the remaining invertebrate TLRs with differences only found in the strength of their relationship to vertebrate TLR4. Similar to the phylogenetic reconstructions of the TLR family restricted to the TIR domain, ML and ME reconstructions agree on two invertebrate clusters: the first consisting of *Anopheles gambiae* a, TLR1b, Toll, and TLR5b and *Drosophila melanogaster* , Toll4, MstProx, and Tehao; and the second consisting of: *Anopheles gambiae* , TLR7, TLR8, TLR10, and TLR11 and *Drosophila melanogaster* Wheeler, TLR7, and Tollo. The only difference in configurations is the placement of and *Caenorhabditis elegans* with the ME reconstruction grouping it in the former cluster whereas the ML reconstruction places it within the latter. In the ME reconstruction the first cluster is modest well supported while the second cluster occurs with strong boot-strap support. In the ML reconstruction, the first cluster is strongly supported while the second cluster shows moderate support. Moderate support is observed in the ME reconstruction for grouping TLR4 with the invertebrate TLRs. This topology is observed in the ML reconstruction but is supported only weakly.
